# Prevalence and Association of Thyroid Dysfunction With Diabetes Mellitus in a Tertiary Care Hospital: A Retrospective Study

**DOI:** 10.7759/cureus.79855

**Published:** 2025-02-28

**Authors:** Vignesh Babu K, Pavalaveelzi CM, Kuzhandai Velu V, Yuvaraj SS

**Affiliations:** 1 Biochemistry, Arunai Medical College and Hospital, Tiruvannamalai, IND

**Keywords:** glycemic status, hyperthyroidism, hypothyroidism, thyroid function test, type 2 diabetes mellitus

## Abstract

Background: Thyroid dysfunction and diabetes mellitus are endocrine disorders that are closely associated with insulin resistance. Thyroid dysfunction has been found to be more common in patients with diabetes compared to those without. In India, thyroid dysfunction is common among patients with diabetes, with a high prevalence found in Tamil Nadu, where subclinical hypothyroidism also contributes significantly.

Aim: This study aims to determine the prevalence and association of thyroid dysfunction among patients with type 2 diabetes mellitus.

Methodology: A retrospective cross-sectional study was conducted from August 2023 to August 2024. A total of 91 patients with diabetes were screened for thyroid dysfunction. Data on demographics, comorbidities, duration of diabetes, glycated hemoglobin (HbA1c), and thyroid function (triiodothyronine, thyroxine, and thyroid-stimulating hormone) were collected. Statistical analysis was performed to identify the association among thyroid dysfunction, glycemic control, and diabetes mellitus duration.

Results: Thyroid dysfunction was identified in 21 (23.1%) of the study population, with 19 (20.9%) patients experiencing hypothyroidism and two (2.2%) patients experiencing hyperthyroidism. The prevalence was found to be high in women. Subjects with diabetes for more than five years had a high prevalence of thyroid dysfunction. HbA1c did not show any significant correlation (p = 0.327).

Conclusion: This study shows the prevalence of thyroid dysfunction, especially hypothyroidism, among patients with diabetes. It also highlights the importance of screening for thyroid function in patients who have had diabetes for more than five years. Early identification and management of thyroid dysfunction may improve glycemic control and reduce the risk of long-term complications.

## Introduction

Thyroid dysfunction and diabetes mellitus (DM) are closely associated endocrine disorders because they can exacerbate one another [1]. Thyroid issues are far more common in patients with diabetes than in those without [2]. In India, 14.7% of individuals with DM, including those with type 1 and type 2 DM, have thyroid abnormalities [3]. In Tamil Nadu state, it was found that 21.5% of those with type 2 DM had thyroid dysfunction, with subclinical hypothyroidism making up the majority (12.4%) [4]. Thyroid issues and DM are more closely related when certain risk factors are present, such as female gender, central obesity, a longer course of DM, and higher glycated hemoglobin (HbA1c) readings, signifying inadequate glycemic control [5-7]. Rong et al. conducted a meta-analysis of 36 studies to study the association between the two endocrine disorders, and the results revealed that prevalence increased with higher age (> 60 years of age with female predilection) [6].

The thyroid hormone is essential for controlling the metabolism of glucose. Because they both increase insulin resistance, hypothyroidism and hyperthyroidism are linked to poor glycemic management. Diabetes can change the function of the thyroid-stimulating hormone (TSH) and affect peripheral tissues’ conversion of triiodothyronine (T3) to thyroxine (T4), resulting in insulin resistance and hyperinsulinemia. These cause the thyroid tissue to proliferate more, resulting in goiter [8]. According to studies, a malfunction in the hypothalamic-pituitary-thyroid axis may be the cause of aberrant thyroid hormone levels [9]. Low thyroid hormone levels in hypothyroidism affect pancreatic β cell activity, hindering the absorption of glucose from the gut. They also reduce peripheral glucose uptake because of increased insulin resistance and decreased insulin sensitivity, as well as gluconeogenesis in skeletal muscles [10]. Meanwhile, in hyperthyroidism, the insulin signaling pathway is impaired, leading to insulin resistance and suspension of hepatic glucose [11]. Oxidative stress is commonly seen in both DM and thyroid disorders. In hypothyroidism, mitochondrial respiratory chain dysfunction causes increased free radical release [12]. Because the thyroid hormone affects lipid metabolism, lower TSH levels can lead to increased total cholesterol and low-density lipoprotein (LDL) levels [13]. A low level of metabolism is usually associated with weight gain and central obesity development. Hypothyroidism affects blood glucose levels and has a deleterious impact on diabetic micro- and macrovascular complications. Vemula et al. conducted a systematic review revealing that hypothyroidism is positively linked with diabetic nephropathy, cardiovascular complications, peripheral neuropathy, and retinopathy [14]. Even subclinical hypothyroidism is associated with diabetic nephropathy and retinopathy [15].

Thyroid dysfunction screening is good for individuals with diabetes because hypothyroidism has several detrimental effects on diabetes. Early diagnosis can aid in suitable and timely management, reduce insulin resistance, and establish adequate glycemic control [16]. It is essential to regularly check for thyroid dysfunction to avoid or postpone microvascular and macrovascular complications of diabetes. Hence, this study aims to determine the association between thyroid dysfunction and patients with type 2 DM in a tertiary care hospital in the northern district of Tamil Nadu, India.

## Materials and methods

A hospital-based cross-sectional study was conducted at Arunai Medical College and Hospital, a tertiary care hospital in Tiruvannamalai, Tamil Nadu. Data from individuals with diabetes who had undergone thyroid screening tests between August 2023 and August 2024 were examined. Following approval by the institutional ethics committee, the central clinical biochemistry laboratory provided the necessary data using the universal sampling approach. Age, gender, length of diabetes, comorbidities (dyslipidemia and hypertension), medication options, and biochemical parameters including fasting blood glucose (glucose oxidase-peroxidase (GOD POD method)), HbA1c (immuno-turbidity), and total thyroid function test (T3, T4, TSH) (chemiluminescence immunoassay (CLIA)) were collected using a semi-structured questionnaire.

Data on a total of 136 patients with diabetes screened for thyroid dysfunction were extracted from the laboratory information system. After excluding records with missing data, known cases of thyroid disorder, critically ill patients, and any other endocrine disorder that affects thyroid disorder, a total of 91 patients’ data were entered into Microsoft Excel (Microsoft Corporation, Redmond, Washington, United States) and coded for data analysis. Data analysis was conducted using IBM SPSS Statistics for Windows, Version 26 (Released 2019; IBM Corp., Armonk, New York, United States). Descriptive data were expressed in terms of means and standard deviations. For inferential statistics, to find the association between thyroid disorder and DM, chi-square/Fisher’s exact test and t-test were used.

Operational definition

According to the American Diabetic Association’s guidelines, HbA1c of less than 5.7% is considered nondiabetic, 5.8-6.4% is considered prediabetic, and > 6.5% is considered diabetic [17]. According to the Diabetic Association of India, patients with diabetes are classified based on an HbA1c value of less than or equal to < 7% in the good glycemic control group and on a value of > 7% in the poor glycemic group [18].

Regarding thyroid disorders, an euthyroid patient is defined as someone whose TSH levels are between 0.45 and 4.5 mU/L; a patient with subclinical hypothyroidism is defined as someone whose TSH levels are between 4.5 and 10 mU/L (in newly detected cases); and a patient with overt hypothyroidism is defined as someone whose TSH levels are more than 4.5 mU/L (in old cases) and > 10 mU/L (in new cases). A TSH level of less than 0.45 indicates hyperthyroidism [19].

## Results

The gender distribution was predominantly female at 61.5%. The percentage of male patients was 38.5%. Additionally, participants’ mean age was 56.53 ± 13.86 (Table 1). Patients had experienced varying durations of diabetes, from one year to > 10 years. The majority of patients fell under the < 5 years category (53.9%). Patients with diabetes had hypertension and dyslipidemia as comorbid conditions and were treated predominantly with oral antidiabetic drugs (68.1%). Participants were subgrouped into glycemic control groups: good, 31 (34%), and poor, 60 (66%), respectively (Table 1).

**Table 1:** Descriptive data of baseline parameter in the study group (N = 91) HbA1c: glycated hemoglobin

Variable	Categories	Frequency (%) N = 91
Gender	Male	35 (38.5%)
	Female	56 (61.5%)
Duration of diabetes	< 5 years	49 (53.9%)
	5 to 10 years	33 (36.3%)
	>10 years	9 (9.8%)
Thyroid dysfunction	Euthyroid	70 (76.9)
	Hypothyroidism	19 (20.9%)
	Hyperthyroidism	2 (2.2%)
Comorbidities	Hypertension	41 (45.1%)
	Dyslipidemia	36 (39.6%)
	Both	15 (15.4%)
Treatment	Insulin	8 (8.8%)
	Oral antidiabetic drugs	62 (68.1%)
	Both	21 (23.1%)
Glycemic control	Good (Hba1c <7)	31 (34%)
	Poor (Hba1c >7)	60 (66%)
Mean ± SD		
Age (years)		56.53 ± 13.86
HbA1c		7.53 ± 1.32

The proportion of thyroid dysfunction was 23.1% (21 patients), out of which 19 patients were diagnosed with hypothyroidism and two with hyperthyroidism. The female diabetic population was more affected by thyroid dysfunction compared with male patients. The mean HbA1c value was found to be 7.53 ± 1.32 among the study population. Laboratory values were compared between good and poor glycemic control groups (Table 2) using the student t-test. HbA1c, TSH, and fasting blood glucose were found to be statistically significant (p-value < 0.05).

**Table 2:** Comparison of biochemical parameters between good and poor glycemic control groups

Parameter	Good glycemic control (HbA1c < 7) (n = 31) Mean ± SD	Poor glycemic control (HbA1c ≥ 7) (n = 60) Mean ± SD	P-value
Triiodothyronine (T3) (ng/dL)	115.32 ± 32.39	110.74 ± 28.36	0.488
Thyroxine (T4) (μg/dL)	8.01 ± 2.26	8.18 ± 1.89	0.716
Thyroid-stimulating hormone (TSH) (µIU/ml)	7.66 ± 10.34	3.44 ± 5.46	0.012*
Fasting blood glucose (mg/dl)	150.48 ± 38.2	229.45 ± 94.87	0.001*
Glycated hemoglobin (HbA1c) (%)	5.93 ± 0.45	9.13 ± 2.19	0.001*

Table 3 shows the association between the duration of DM and thyroid dysfunction. A chi-square test was performed. The association was found to be statistically significant (X² = 11.536; p = 0.003). This implied that if the duration of diabetes moves beyond five years, the chances of developing thyroid dysfunction increase significantly. The results of a chi-square test of the association showed no significant difference (X² = 2.238; p = 0.327) (Table 4).

**Table 3:** Association of duration of diabetes with thyroid dysfunction

Duration of diabetes	Euthyroid N (%)	Thyroid dysfunction N (%)	X^2^	P-value
< 5 years	44 (62.86%)	5 (23.81%)	11.536	0.003*
5–10 years	19 (27.14%)	14 (66.67%)		
> 10 years	7 (10.00%)	2 (9.52%)		

**Table 4:** Association of glycemic control with thyroid dysfunction in patients with diabetes HbA1c: glycated hemoglobin

HbA1c	Euthyroid N (%)	Hypothyroidism N (%)	Hyperthyroidism N (%)	X^2^	P-value
Poor control HbA1c ≥ 7	49 (70)	10 (52.63)	1 (50)	2.238	0.327
Good control HbA1c < 7	21 (30)	9 (47.37)	1 (50)		

## Discussion

Thyroid dysfunction is a significant public health concern that affects individuals with DM. Diabetes and thyroid issues have a complex relationship that influences several factors. Sam et al. proposed insulin resistance as a cause of DM and thyroid dysfunction, with high insulin levels potentially altering thyroid tissue [20]. Additionally, there is a reciprocal relationship between the two conditions, with uncontrolled diabetes aggravating thyroid dysfunction and thyroid abnormalities negatively affecting glycemic control. Autoimmune processes are also significant in type 1 diabetes, where autoimmunity-induced thyroid disease is a common comorbidity [21].

Thyroid dysfunction was observed to affect 21 (23.1%) patients with diabetes in the current study. Comparable research by Thankappan PK in Kerala found that 26.7% of people had thyroid malfunction [22]. Further, according to research by Asuti S. et al. conducted at a tertiary care hospital, 23.6% of patients with type 2 diabetes had thyroid abnormalities [23]. Similar findings were found in a study by Khassawneh AH et al., which showed that 26.7% of patients with diabetes had thyroid disorders [24]. In a related investigation, Palma et al. discovered that 14.7% of patients with diabetes had thyroid dysfunction [3]. A study conducted by Yadav et al. to determine the prevalence of thyroid disorders among patients with diabetes revealed that 20.1% of patients had thyroid dysfunction [25]. In all these studies, hypothyroidism was the most common disorder, followed by subclinical hypothyroidism and hyperthyroidism.

According to the current study’s findings, thyroid dysfunction is substantially more prevalent in female patients with diabetes than in male patients. Perros et al. discovered that thyroid disorder was present in 31.4% of female patients with diabetes and 6.9% of male patients with diabetes [26]. Studies have consistently demonstrated that thyroid disorders are more prevalent in women than in men [26]. These findings highlight the need for gender-specific approaches to the early identification and management of thyroid gland disorders in patients with diabetes.

Thyroid dysfunction seems to be positively correlated with the length of having diabetes. According to our study, 76.19% of patients with diabetes lasting more than five years had thyroid dysfunction. More than 60% of individuals with thyroid dysfunction had a prior diagnosis of DM that lasted more than five years, according to studies. People with diabetes who had the disease for an average of 10.92 years had a significant prevalence of thyroid abnormalities [27]. Likewise, low TSH levels were reported in individuals with diabetes for a period of five years or longer [28]. However, studies have reported no significant association between diabetes duration and thyroid dysfunction [23]. These findings indicate the need for further investigation to clarify the relationship and its implications for patient management.

According to the current study, hypothyroidism accounted for more than 90% of thyroid conditions in the diabetic population. Mehalingam et al. (2020) found that 20.7% of the participants had either clinical or subclinical hypothyroidism, accounting for 22.5% of thyroid dysfunction [7]. Furthermore, most patients with diabetes have subclinical hypothyroidism, according to the literature [29]. These results highlight how crucial it is to regularly test individuals with diabetes for hypothyroidism, especially in its subclinical form, to guarantee early identification and suitable treatment.

Hence, this study concludes that patients with diabetes face an increased risk of developing thyroid dysfunction, which majorly increases with the duration of their diabetes (i.e., more than five years). This signifies the importance of early clinical screening for thyroid dysfunction to initiate early treatment.

Limitations

This study has certain limitations. First, this was a retrospective study, so only existing data could be analyzed. Thus, there may be missing data that were not taken into account. Additionally, the small sample size limits the generalizability of the findings to a larger population. Because this is a retrospective study, certain aspects regarding physical activity, drug intake, and dietary habits could not be assessed.

Recommendations

To facilitate early identification and treatment, it is advised that patients with diabetes, particularly those who are female and have had the disease for a longer period, undergo regular thyroid dysfunction screenings. Thyroid function testing must be incorporated into routine diabetes treatment procedures by healthcare systems, especially for high-risk individuals. To treat the intricate interactions between these disorders, multidisciplinary methods combining endocrinologists and diabetologists are essential. The significance of identifying thyroid dysfunction symptoms and implementing healthy lifestyle habits should be emphasized in patient education programs.

## Conclusions

Thyroid dysfunction and type 2 DM were significantly correlated among patients. A total of 23.1% of people had thyroid malfunction, with hypothyroidism being the most prevalent kind. The study also found that people with diabetes who were female and had the disease for a longer period were more likely to have thyroid abnormalities. Although glycemic control did not show a statistically significant association with thyroid dysfunction, poor glycemic control was prevalent in the majority of the study population.

It is crucial to regularly evaluate patients with diabetes for thyroid abnormalities because thyroid dysfunction has a negative impact on glycemic management and diabetes complications. Improvement of metabolic control and prevention or delay of diabetes-related micro- and macrovascular problems can be achieved through early identification and prompt treatment of thyroid dysfunction, especially hypothyroidism. The intricate relationship between diabetes and thyroid diseases should be further investigated in future research with bigger, more varied population groups and prospective designs to provide better insights into patient management.
